# Current implications and challenges of artificial intelligence technologies in therapeutic intervention of colorectal cancer

**DOI:** 10.37349/etat.2023.00197

**Published:** 2023-12-27

**Authors:** Kriti Das, Maanvi Paltani, Pankaj Kumar Tripathi, Rajnish Kumar, Saniya Verma, Subodh Kumar, Chakresh Kumar Jain

**Affiliations:** University of Campania, Italy; ^1^Department of Artificial Intelligence and Precision Medicine, School of Allied Health Sciences and Management, Delhi Pharmaceutical Sciences and Research University, New Delhi 110017, India; ^2^Department of Biotechnology, Jaypee Institute of Information Technology, Noida 201309, Uttar Pradesh, India; ^3^Department of Medical Laboratory Technology, School of Allied Health Sciences, Delhi Pharmaceutical Sciences and Research University, Delhi 110017, India

**Keywords:** Artificial intelligence, machine learning, deep learning, colorectal cancer, drug discovery

## Abstract

Irrespective of men and women, colorectal cancer (CRC), is the third most common cancer in the population with more than 1.85 million cases annually. Fewer than 20% of patients only survive beyond five years from diagnosis. CRC is a highly preventable disease if diagnosed at the early stage of malignancy. Several screening methods like endoscopy (like colonoscopy; gold standard), imaging examination [computed tomographic colonography (CTC)], guaiac-based fecal occult blood (gFOBT), immunochemical test from faeces, and stool DNA test are available with different levels of sensitivity and specificity. The available screening methods are associated with certain drawbacks like invasiveness, cost, or sensitivity. In recent years, computer-aided systems-based screening, diagnosis, and treatment have been very promising in the early-stage detection and diagnosis of CRC cases. Artificial intelligence (AI) is an enormously in-demand, cost-effective technology, that uses various tools machine learning (ML), and deep learning (DL) to screen, diagnose, and stage, and has great potential to treat CRC. Moreover, different ML algorithms and neural networks [artificial neural network (ANN), k-nearest neighbors (KNN), and support vector machines (SVMs)] have been deployed to predict precise and personalized treatment options. This review examines and summarizes different ML and DL models used for therapeutic intervention in CRC cancer along with the gap and challenges for AI.

## Introduction

Cancer is the second leading cause of death across the globe [1]. In terms of mortality and morbidity, Global Cancer Statistics 2020 shows that, out of 36, colorectal cancer (CRC) is the third most common cancer in the population. Worldwide, it affects equally to both men and women equally. Every year more than 1.85 million cases of CRC have been reported and 20% of them have metastatic disease at presentation. The estimated number of deaths by 2023 for CRC is 52,550 [2]. CRC is the third most common type of cancer in both sexes and demands an early diagnosis and treatment to save the lives of many [3]. It begins with the formation of tiny clusters called polyps. Some of these polyps turn malignant resulting in CRC over a period of 10–15 years. Males are more likely to be associated with CRC. Family history of CRC in correspondence to age and the extent of its effect on the relatives play a role in 10–20% of all CRC patients. Individuals with older age and lasting bowel inflammation are at higher risk [4]. Researchers of various domains are discovering different approaches to tackle this disease.

Artificial intelligence (AI) can be used to improve CRC treatment and treatment methods. With the huge amount of data generated by medical imaging, computed tomography, histopathology evaluation, etc. comes the use of AI [5].

In addition, machine learning (ML) algorithms can be used to create predictive models to help clinical decision-making without any prior explicit programming [5]. Many modalities and sub-specialties of AI show promise for the application of predictive studies, distribution, and prevalence of CRC and thus enable personalized approaches in drug discovery uplifting precision medicine and subsequently clinical practices [6, 7]. Drug development is inevitably a delicate and challenging procedure that puts a strain on productivity and research and development (R&D) costs.

The diagnosis and categorization of diseases and their subtypes among patients are made possible by a variety of deep learning (DL) and statistical techniques that depend on data interpretation.

**Figure 1:**
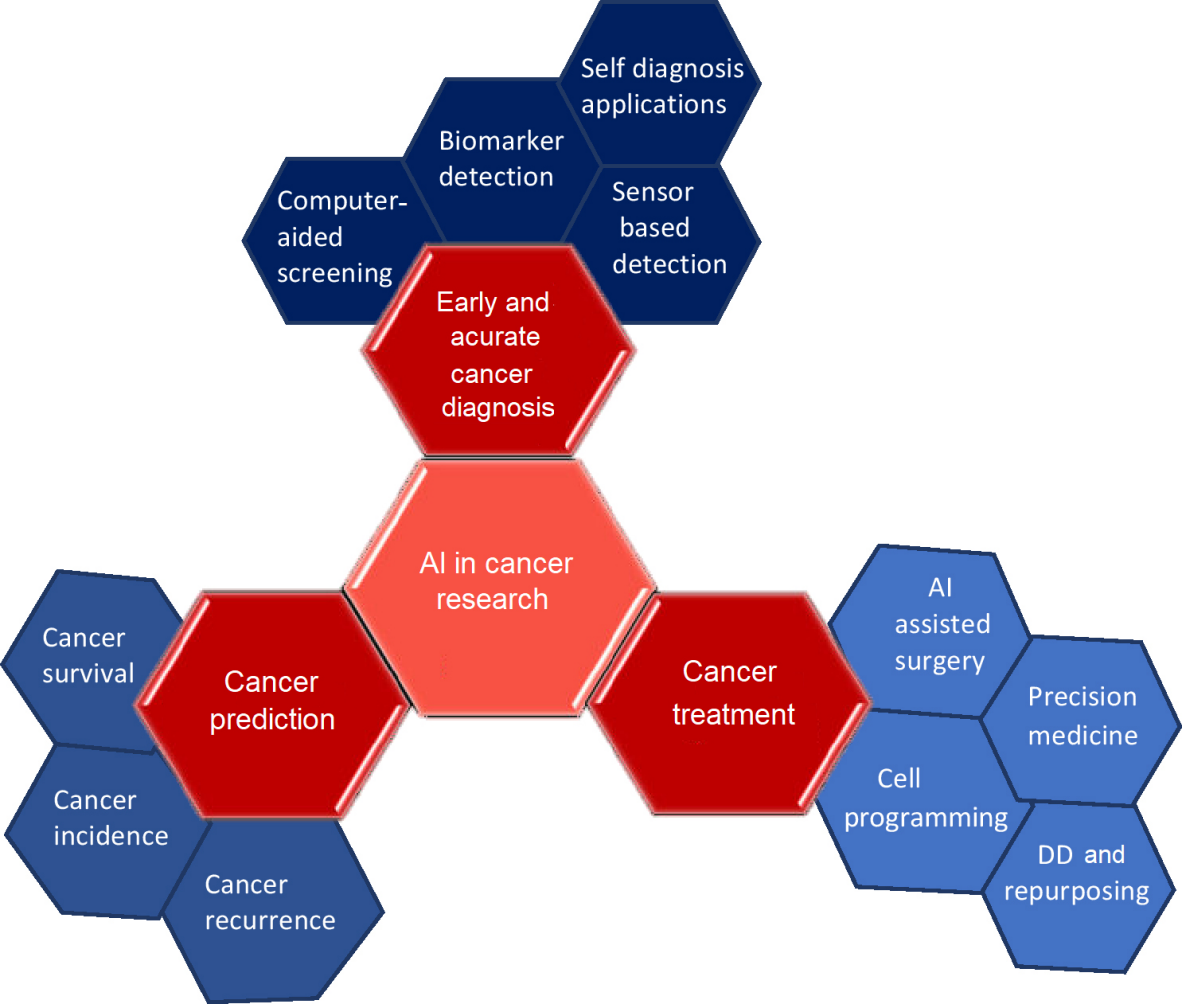
How and where AE in cancer research is being used

To detect disease targets quickly and accurately, ML, feature-finding, and clustering techniques are useful. The application of statistical analysis on big data, experimental data, and data mining methods, together with neural networks, improves capacity for *de novo* drug designing (DD). The use of existing drugs for new therapeutic applications is drug repurposing also called repositioning. The use of metformin, a type 2 diabetes medication showed reduced chances of developing CRC in 47,000 participants [8]. The area of precision medicine is advanced by drug repurposing and combination therapy based on numerous genomic markers and increased patient information (Figure 1) [6]. Based on the literature survey, this review provides a description of CRC and the performance of various AI-based models in chemotherapy and neoadjuvant chemoradiotherapy (nCRT) for its therapeutic intervention. It also shows the application of existing AI-backed computational tools in the domain of drug discovery and development processes to enhance treatment options for colon cancer and other disease conditions. Finally, it spreads light on the challenges that lie ahead of AI for drug repurposing, *de novo* DD, and therapeutic options for various disease conditions including CRC.

## Functionalities of AI

**Figure 2:**
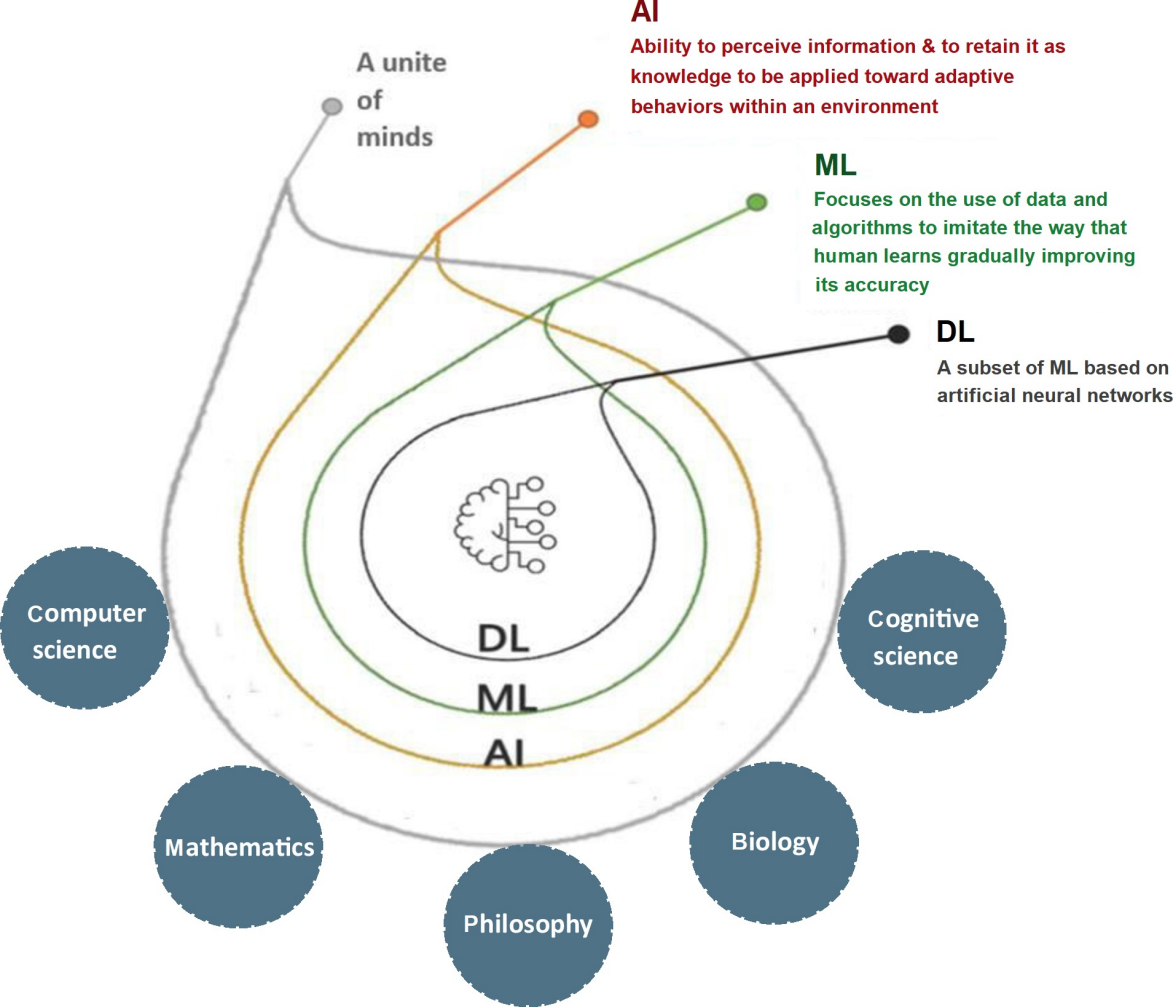
Applications of AI in different disciplines, utilizing DL and ML

A subfield of computer science called AI encompasses various fields, including mathematics, logic, philosophy, psychology, cognitive science, and biology. AI refers to intelligent technology that has been artificially created to mimic humans. This AI is incorporated into a computer system known as an AI system, which eventually serves as a thinking machine. The three characteristics of the AI system are intelligence, intentionality, and adaptability. A variety of strategies can be used to create an AI system that effectively performs human tasks. AI supports the system’s decision-making process and aids in outcome forecasting. To advance current technology or develop new ones, AI combines different ML algorithms and neural networks (Figure 2).

### Basics of ML

**Figure 3:**
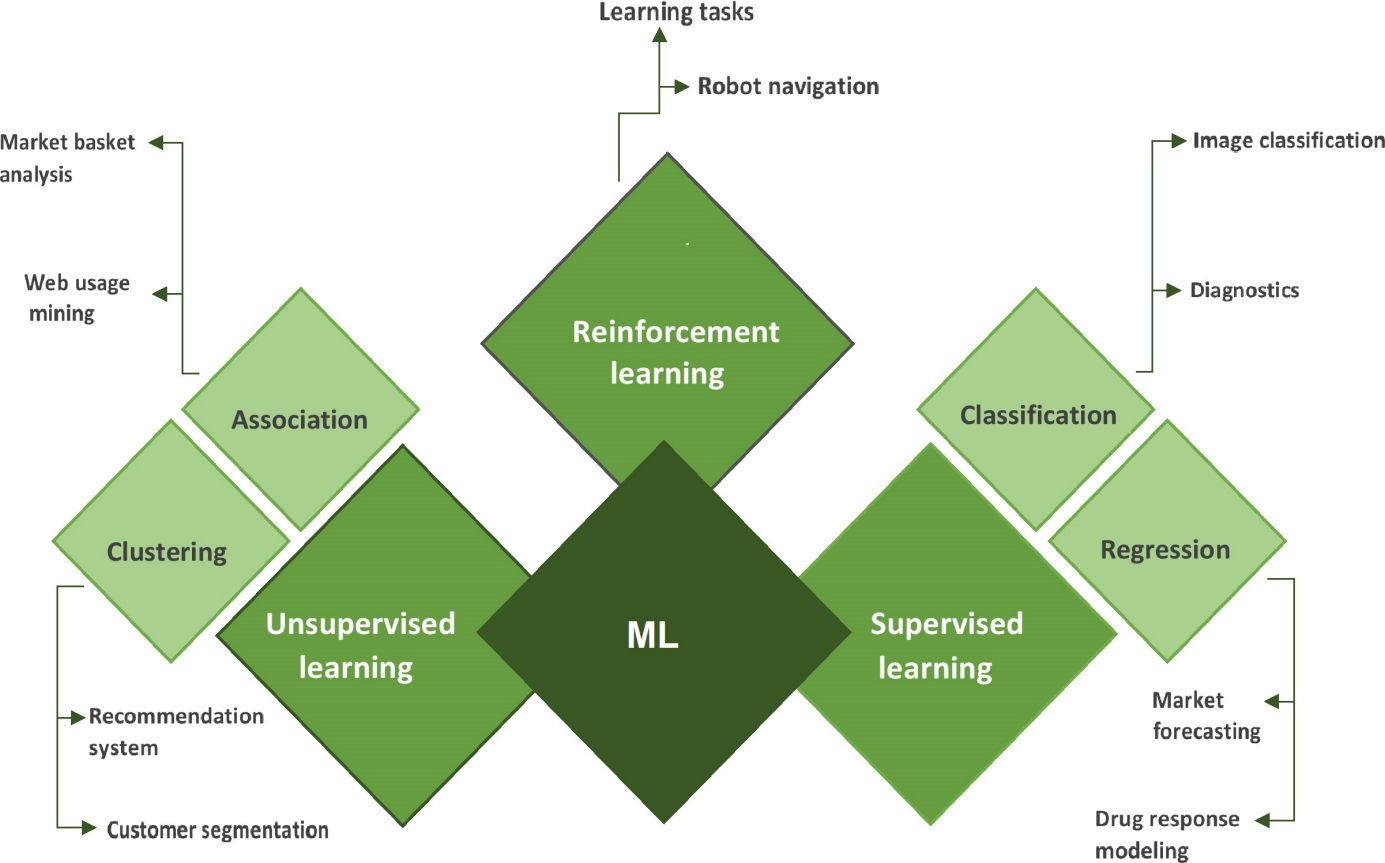
Categorization of ML algorithms with its subtypes and their applications

A subtype of AI called “ML” enables computers to learn from their surroundings automatically and without human involvement, which suggests that they are developing their decision-making abilities. ML employs a number of algorithms and strategies to classify and enhance the data to make better predictions. In the medical sciences, ML techniques are now applied for the detection and categorization of distinct tumor forms. First, ML algorithms look for patterns, and then they take actions based on those patterns [9, 10]. ML can be primarily divided into three types: supervised learning, unsupervised learning, and reinforcement learning (Figure 3).

In supervised learning, both the input and output data are provided by the trainer. It is a kind of ML in which the data has been labeled so that the machine may discover and build patterns between the input and output data. By identifying the pattern, it can learn how to classify or categorize the data [11]. Supervised learning is of two types viz (A) classification and (B) regression.

Classification is a type of supervised learning that is used to predict/classify discrete values such as male or female, yes or no, malignant or benign, etc. Some classification algorithms under supervised learning are decision trees, random forest (RF), logistic regression (LR), support vector machines (SVMs), etc. [12].

Similarly, regression is another type of supervised learning used to predict continuous values. Some regression algorithms under supervised learning, i.e., regression trees, linear regression, non-linear regression, polynomial regression, etc. [12, 13].

Unsupervised learning is a type of ML in which algorithms may uncover previously undiscovered patterns in unlabelled datasets and provide the desired output without any external help. Unlabelled datasets are analyzed and clustered using ML techniques [11]. Unsupervised learning is of two types viz (A) clustering and (B) association. Clustering is a technique for organizing data points into various clusters made up of related data points. Finding correlations between variables in a large database is done using the unsupervised learning technique of association.

Some algorithms are under unsupervised learning, i.e., k-means clustering, apriori algorithm, hierarchal clustering, independent component analysis, k-nearest neighbors (KNN), and principle component analysis (PCA).

Reinforcement learning is an ML strategy that relies on feedback, in which an agent learns automatically utilizing feedback rather than labeled data. The agent is necessitated to learn exclusively from its own experience because there isn’t any labeled data.

#### SVM

**Figure 4:**
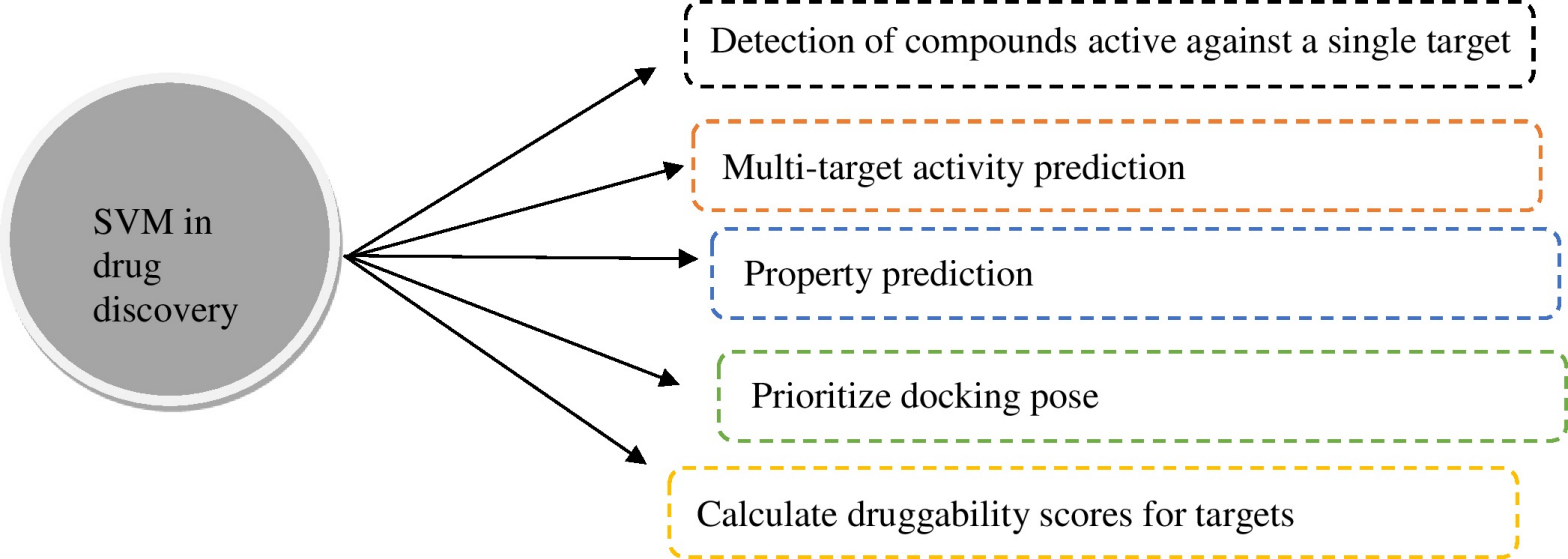
Applications of SVM in drug discovery

Researchers have used ML techniques for datasets for the diagnosis and treatment of cancer. Although there are many methods proposed for classification, SVM is the most popular due to its strong mathematical foundation based on structural risk minimization, statistical learning theory, and its accurate performance. SVM is a pattern recognition tool [14]. SVM is being used in many ways in the field of drug discovery (Figure 4) [15].

For linear classification, the decision function can be formulated as equation 1 (Eqn. 1). For non-linear classification, the decision function can be formulated as Eqn. 2.

Eqn. 1:

**Figure:**
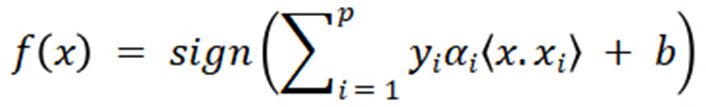


Eqn. 2:

**Figure:**
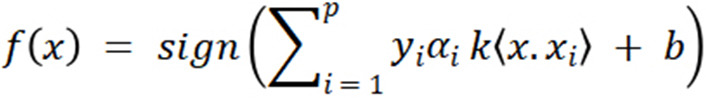


Where, *k* = kernel function; *x_i_* = n-dimensional vector; *y_i_* = its label; and *a_i_* = Lagrange multipliers.

#### Naive Bayes classifier model

Naive Bayes classifier (NBC) are simple “probabilistic classifiers” based on Bayes theorem with naive (strong) independence assumption between the features (Eqn. 3). Naive Bayes has been used to diagnose CRC by identifying the origin of tumor cells using RNA sequence data [16].

Eqn. 3:

**Figure:**
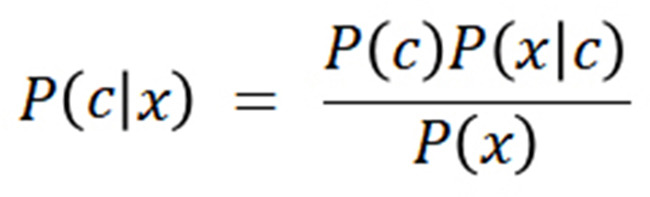


Where, *x* = attributes; *c* = class; *P*(*c*|*x*) = probability of “*c*” being true, given that “*x*” is true; *P*(*x*|*c*) = probability of “*x*” being true, given that “*c*” is true; *P*(*c*) = probability of “*c*” being true; and *P*(*x*) = probability of “*x*” being true.

Using Bayesian probability, the above Eqn can be written as Eqn. 4.

Eqn. 4:

**Figure:**
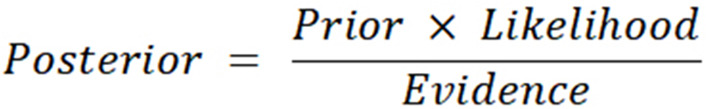


#### LR

LR is the practical application of AI for disease prognosis and management. LR models predict the probability of values ranging from 1 and 0. It is mostly applied to categorical data [17]. For example, if the cancer is malignant (1) or not (0). LR can be represented by Eqn. 5:

Eqn. 5:

**Figure:**
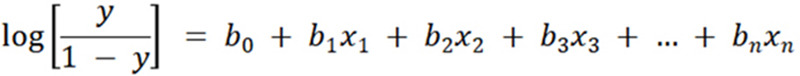


Where, *y* = predicted output; *b*_0_ = intercept; *x* = input value; and *b*_1_ = co-efficient of the input value 𝑥 (single value).

### DL

DL is an ML technique that trains a computer to filter inputs through layers as it gains the ability to predict and categorize data. It basically consists of a neural network with three or more layers. These neural networks attempt to mimic how the human brain functions [12, 18]. This has been classified broadly into convolutional neural networks (CNNs), artificial neural networks (ANNs), and recurrent neural networks (RNNs).

CNNs are a specific type of neural network that is mostly used for object recognition, image clustering, and image classification [19], while the ANNs mimic the biological neural networks of the human brain and are typically comprised of three layers, i.e., (A) the input layer which accepts input from the programmer in a variety of formats; (B) hidden layer: these layers are situated in-between the input and output layers and it performs all calculations to reveal hidden characteristics and patterns; and (C) output layer: this layer is used to convey the output after the input has undergone several transformations utilizing hidden layers.

RNNs are a specific kind of ANN that is mostly used in speech recognition and natural language processing (NLP). Because their mathematical processes are performed sequentially, RNNs get their name [19, 20].

## Steps of drug discovery and AI

The long and difficult process of finding new drugs can be roughly broken down into the following stages: (A) target identification; (B) target validation; (C) lead identification; (D) lead optimization; (E) product characterization; (F) formulation and development; (G) pre-clinical research; (H) investigational new drug; (I) clinical trials; (J) new drug application; and (K) approval [21]. Regarding a certain ailment, it is required to first determine the target. In the following phase, hit identification, molecules in molecular libraries are identified using techniques including combinatorial chemistry, high-throughput screening, and virtual screening. In a clinical study, the medication candidate is finally given to patients after passing all preclinical tests satisfactorily.

The medication must proceed sequentially through each of the three stages of this process. Phase I entails doing drug efficacy tests on a handful of individuals with the specified ailment; phase II entails running drug safety tests on a smaller number of human subjects; and phase III entails performing effectiveness tests on a wider range of patients. If the drug candidate’s safety, as well as effectiveness, are shown during the clinical phases, agencies like the Food and Drug Administration (FDA) review the substance for authorization and marketing. A traditional drug discovery pipeline is thought to cost an average of 2.6 billion dollars, and it may take up to 12 years to accomplish [22, 23].

**Figure 5:**
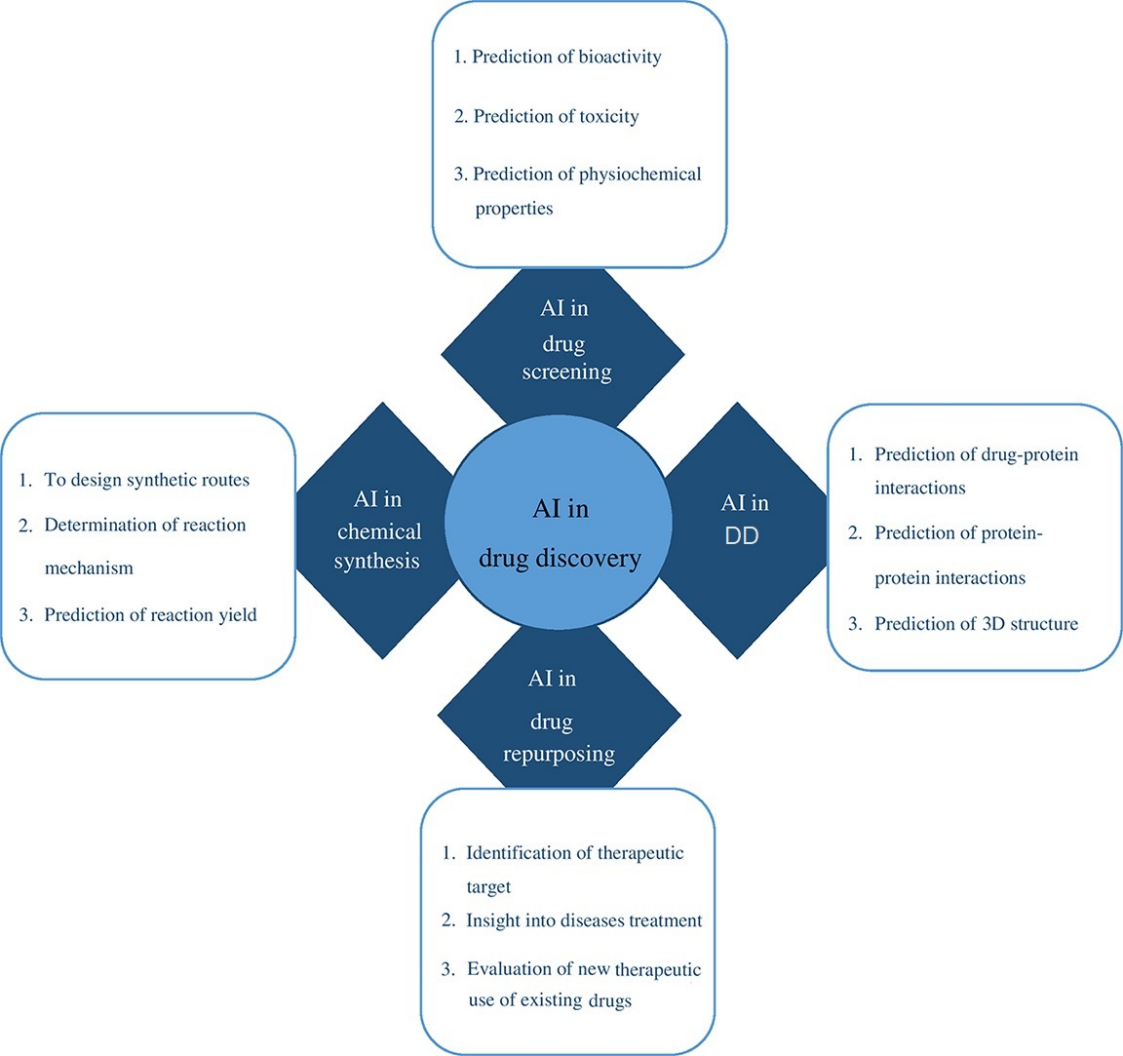
AI in drug screening, DD, drug repurposing, and chemical synthesis

The main concerns for all pharmaceutical companies are how to save expenses and advance initiatives. To increase productivity and cut costs, AI-based computational tools are being used at various phases of the drug discovery process (Table 1). These include cell classification and real-time image-based cell sorting, as well as computer-aided organic synthesis, design of new molecules, assay development, and prediction of the three-dimensional (3D) structures of target proteins, among many other uses (Figure 5). In general, AI can automate and optimize these time-consuming processes to dramatically speed up R&D medication development [24, 25]. Also, AI is used to coordinate, operate, and recruit participants for clinical trials, frequently associated with improved patient monitoring during clinical trials or with medical equipment that can access specific patient data and guide medical decisions [26, 27].

**Table 1:** Computational tools for drug discovery: AI-based

**Tools**	**Purpose**	**References**
DeepChem	Drug discovery task prediction	[28]
DTI-CNN	DL based drug-target interaction prediction	[29]
ORGANIC	Molecular generation tool with desired properties	[30]
Chemputer	Chemical synthesis reporting procedure	[31]
DeltaVina	Rescoring protein-ligand binding affinity: scoring	[32]
DeepCPI	Drug–protein interaction prediction	[33]
PotentialNet	A CNN graph-based ligand-binding affinity prediction	[34]
DeepNeuralNet-QSAR	Prediction of molecular activity	[35]
Hit Dexter	Prediction of molecules responding to biochemical assays	[36]
DeepTox	For toxicity prediction	[37]
PPB2	Polypharmacology prediction	[38]
SCScore	For evaluation of the synthesis complexity of a molecule	[39]
NNScore	Protein-ligand interaction scoring study	[40]
SIEVE-Score	Structure-based virtual screening	[41]
REINVENT	Molecular *de novo* design based on RNN and RL	[42]

RL: reinforcement learning; DTI-CNN: drug-target interaction-CNN; QSAR: quantitative structure-activity relationship; PPB2: polypharmacology browser 2; SCScore: synthetic complexity score; SIEVE-Score: similarity of interaction energy vector-score; DeepTox: DL for toxicity; NNScore: neutral-network receptor-ligand scoring function

A quick overview of the recent instances of drug development using AI techniques has been discussed as shown in Figure 5. The developing field of AI has garnered limited attention despite its significant expansion. The computational creation of novel structures with desired attributes, known as *de novo* design, is a focal point, particularly starting with fresh chemical matter. Likewise, the related domains of forward prediction and retrosynthesis prediction, seeking to establish how chemical matter designated for experimental research can be synthesized, have also piqued substantial interest. Determining whether a ligand binds to a specific protein target once it has been placed is the logical next step, and target prediction *in silico* and docking (and related techniques) have been active research fields for decades [43]. In terms of predicting ligand-protein interactions, methods like DL have a somewhat good effect on improving numerical measurements of performance (often marginally). This hasn’t always been the case, though, as evidenced by a recent large-scale study that found no benefit to DL in terms of performance. Also, special attention must be paid to the model performance measurements employed in this context and if they reflect a pertinent metric capable of detecting both significant and practically applicable changes in model quality [44].

### AI in drug discovery

The process of creating efficient new pharmaceuticals is the most complicated part of the medication development process. The techniques that incorporate AI have evolved into flexible toolkits that can be used widely in several stages of drug development, including the identification and validation of drug targets, the design of new drugs, drug repurposing, improving R&D efficiency, the analysis of biomedicine data, and the improvement of the decision-making process to enroll patients in clinical trials [21]. While addressing the inefficiencies and uncertainties brought on by the conventional techniques of drug development, these potential applications for AI offer the ability to reduce bias and human meddling in the process [45, 46].

Further applications of AI in drug development include pharmacological qualities, protein features and efficacy, drug combination and DTI, drug repurposing, drug synergism/antagonism prediction, and prediction of practical synthetic methods for drug-like compounds [47]. Finding new pathways and targets using omics research is made feasible by the development of novel biomarkers and therapeutic targets, the creation of personalized medicine based on omics markers, and the discovery of connections between drugs and illnesses.

When it comes to suggesting powerful medication ideas and correctly anticipating both their qualities and potential toxicity hazards, DL has shown exceptional effectiveness. The analysis of enormous datasets, arduous compound screening while minimizing standard error, and the requirement for major R&D costs and time of over US$ 2.5 billion each decade may all be avoided with the application of AI approaches. With the aid of AI technology, new research may be conducted to aid in the discovery of new drug targets, logical medication design, and drug repurposing [44].

## AI-based therapeutics in CRC

Chemotherapy, nCRT, and more comprehensive methods of treatment are available for CRC. Utilizing AI for CRC treatment, clinicians can choose the best-suited treatment option and increase the effectiveness of treatment by creating a personalized treatment course for each patient [1, 48]. AI-based interventions have been proven a state-of-the-art method to identify the appropriate surgery method, especially in handling complicated situations in CRC patients [49]. Further, these methods have been proven to be indispensable tools in the investigation of the precise stage of heterogeneity level of CRC during its diagnostic and suggest the possible management method. AI and ML present the ability to achieve early detection and diagnosis by precisely detecting polyps and lesions through image analysis. AI plays a promising role in improving accuracy and efficiency, especially in image analysis and molecular profiling [3]. ML identifies CRC biomarkers for non-invasive screening, while neural networks assist in analyzing the histopathologic images and reduce the expertise gaps. AI boosts medical image readability and guides precise robotic surgery, thus benefiting CRC treatment. AI also enhances nCRT, improving CRC treatment and efficacy assessment [50]. The table offers details about research on AI models for CRC treatment in relation to chemotherapy and nCRT (Table 2).

**Table 2:** Recent research on AI models for predicting nCRT and chemotherapy response in the treatment of CRC

**Topic**	**Research**	**Model**	**Performance**	**Year**	**Reference**
nCRT	EUS images of 43 LARC patients as predictive biomarkers Images pre-processed by lee, wiener, median, frost, bilateral, and wavelet filters	LR and SVM	AUC: 0.71 and 0.76 Accuracy: 70.0% and 71.5% Sensitivity: 69.8% and 80.2% (respectively)	2022	[51]
	CT images of 215 LARC patients Images evaluated by filtration histogram texture analysis and fractal dimension	LR	Accuracy: 82% Specificity: 89% Sensitivity: 60%	2021	[52]
	pCR prediction in 282 LARC patients (248 training and 34 validation)	ANN	AUC/accuracy/sensitivity: 0.84/0.88/0.94 respectively	2020	[53]
	pCR prediction in 6,555 non-metastatic cancer patients undergoing radical resection	LR	92.4%/88.2%: With/without—pathological complete response (overall survival rate of 3 years)	2019	[54]
	MRI of 98 patients (53/45: training test/validation set respectively) Image preprocessing by EMLMs and LOG filters	SVM, NN, BN, and KNN	Test (AUC and accuracy): 97.8% and 92.8% Validation (AUC and accuracy): 95% and 90%	2019	[55]
	MRI of 55 LARC patients to predict pCR and pNR rates	RF	0.83: Mean of AUC	2019	[56]
Chemotherapy	Irinotecan drug toxicity prediction in 20 metastatic CRC patients (liver function bloody tests and tumor markers)	SVM	Accuracy: 91%/76%/75% for diarrhea/leukopenia/neutropenia respectively	2019	[57]
	Detection of IC_50_ of a drug Evaluation of QSAR using NMR Analysis of 18,850 organic compounds	KNN, RF, and SVM	Above 63% accuracy	2018	[58]

AUC: area under curve; NN: neural network; BN: Bayesian network; LARC: locally advanced rectal cancer; EUS: endorectal ultrasound; IC_50_: half maximal inhibitory concentration; LOG: Laplacian of Gaussian; NMR: nuclear magnetic resonance; CT: computed tomography; MRI: magnetic resonance imaging; pCR: pathologic complete response; EMLMs: ensemble machine learning models; pNR: pathologic non responder

The following table enlists the FDA-approved individual drugs for CRC treatment. However, the drug combinations are not FDA-approved but the drugs individually are approved by FDA. The list does not include all the drugs and there may be more drugs (Table 3) [59].

**Table 3:** FDA-approved medications for colon and rectal cancer consisting of both generic and brand names

**FDA-approved individual drugs (CRC)**	**Mechanism of action**
Ipilimumab	Prevent inhibition of T-cell mediated immune responses to tumors by binding CTLA-4
Bevacizumab (Mvasi)	Mvasi, a mAb, inactivates serum VEGF treating metastatic CRC
Pembrolizumab	Binds to PD-1 inhibiting its interaction with PD-L1 and PD-L2. Enhances anti-tumor immune response and tumor immune monitoring
Tucatinib	Inhibits HER-2 suppressing tumors by affecting cell proliferation and AKT and MAPK signaling
Ziv-aflibercept (Zaltrap)	Inhibits VEGF-A and PIGF to reduce vascular permeability and inhibit neovascularization. Delay vision loss and advancement of metastatic CRC
Irinotecan hydrochloride	Restricts DNA strands from elegating by attaching to the topoisomerase I-DNA complex and inhibiting its action, which causes fatal double-stranded splits in the DNA. Causes apoptosis as DNA damage is ineffectively repaired
Nivolumab	Restores tumor-specific T-cell response in patients. Binds to PD-1, avert PD-L1 and PD-L2 from blocking the action of T-cells
Ramucirumab	By binding to VEGFR2, it halts the ligands (VEGF-A, VEGF-C, and VEGF-D) from binding to it, blocking VEGF-stimulated receptor phosphorylation and the proliferation, permeability, and migration of human endothelial cells that are later caused by ligands
Cetuximab	Blocks the binding of EGF, which in turn prevents EGFR activation. It also binds selectively to the EGFR and phosphorylates and activates receptor-associated kinases (MAPK, PI3K/AKT, and JAK/STAT)
Regorafenib	At clinically attained concentrations it inhibits the function of VEGFR1, VEGFR2, VEGFR3, RET, PDGFR-alpha, PDGFR-beta, KIT, FGFR1, FGFR2, BRAFV600E, PTK5, TIE2, TrkA, RAF-1, BRAF, DDR2, SAPK2, Eph2A, and *Abl*
Panitumumab	Panitumumab binds primarily to EGFR and prevents ligands from binding to EGFR
Leucovorin calcium	By strengthening the bond between the active metabolite (5-FdUMP) and the enzyme thymidylate synthetase, leucovorin increases the action of fluorouracil

CTLA-4: cytotoxic T lymphocyte antigen-4; mAb: monoclonal antibody; PD-1: programmed death-1; PD-L1: PD-ligand-1; HER-2: human epidermal growth factor receptor-2; AKT: AKT serine/threonine kinase; MAPK: mitogen-activated protein kinase; VEGF-A: vascular endothelial growth factor-A; PIGF: placental growth factor; I-DNA: I-motif DNA; VEGFR2: VEGF receptor 2 ; EGF: epidermal growth factor; EGFR: EGF receptor; PI3K: phosphatidylinositol-4,5-bisphosphate 3-kinase; JAK: Janus kinase; STAT: signal transducer and activator of transcription; RET: Ret proto-oncogene; PDGFR-alpha: platelet derived growth factor receptor alpha; KIT: KIT proto-oncogene, receptor tyrosine kinase; FGFR1: fibroblast growth factor receptor 1; PTK5: protein tyrosine kinase 5; TIE2: tyrosine kinase with immunoglobulin like and EGF like domains 2; RAF-1: Raf-1 proto-oncogene, serine/threonine kinase; BRAF: B-Raf proto-oncogene, serine/threonine kinase; DDR2: discoidin domain receptor tyrosine kinase 2; SAPK2: sucrose non-fermenting-1-related protein kinase 2; Eph2A: ephrin type-A receptor 2; *Abl*: a gene; 5-FdUMP: 5-fluorodeoxyuridine monophosphate; BRAFV600E: B-Raf proto-oncogene; TrkA: tropomyosin receptor kinase A

The names of the drugs are shorthand references to the NCI’s cancer drug information. It’s possible that some drugs used to treat rectal and colon cancer aren’t included here. Some drugs combination used currently for CRC viz capecitabin and oxaliplatin (CAPOX), leucovorin, fluorouracil, and irinotecan (FOLFIRI), FOLFIRI-CETUXIMAB, oxaliplatin, 5-fluorouracil, and folinic acid (FOLFOX), capecitabine and irinotecan (XELIRI), 5-fluorouracil-leucovorin (FU-LV), FOLFIRI-BEVACIZUMAB, and oxaliplatin and capecitabine (XELOX) [59].

## Challenges

Certain components of the drug development process haven’t gained enough attention yet. For instance, it is currently challenging to determine precisely how well a drug candidate binds to its intended protein target [24, 60, 61]. AI and other computational techniques do not currently perform well in this field for several reasons [24, 43, 62].

First off, AI is a data-mining technique. When using AI for data mining, the amount and quality of the available data directly affect how well AI models work [20, 24, 63–65]. Large volumes of training data are necessary for effective DNN training. The creation of transfer learning technology, that applies the lessons it picks up from one activity to another, could be a viable solution to this issue. The second problem is that occasionally the data quality is not good enough for effective AI learning. Biological assays, techniques, or conditions are frequently different from those used to measure experimental data in public databases. A substance can provide completely different results from measurements made using several techniques, which are incomparable. Also, outstanding data could be found in public databases [66].

There have been many unresolved questions such as how AI can be utilized to reliably estimate the binding affinity of a novel drug considering that the scaffold is distinct from the training datasets available. How AI can predict changes in protein structure that can occur at microseconds or even second timeframes? If AI can predict a new drug’s complex physical properties, such as its capacity to pass through the brain-blood barrier (BBB), membrane permeability, etc.? The most important therapeutic targets in drug research would be revealed if AI could predict new G protein-coupled receptors (GPCR) allosteric sites [24]. Hence, selecting high-quality data from the raw inputs is a crucial step before doing specific AI operations. AI could provide the answer by automating data entry as well. Finally, when a 2D interpretation of 3D atomic space occurs for AI computations, crucial 3D target structural information is lost, including the chemical surrounding of the target protein’s ligand binding site, the drug molecule’s conformation, and the protein’s flexibility. Alternatively, proteins and drug molecules could be sampled in varied conformations and states within physiological settings using molecular dynamics (MD) simulations. The effectiveness of this method was recently demonstrated in a study that used AI and MD simulations to examine the ligand specificity of GPCRs. Shortly, it’s possible to get beyond the constraints of binding-affinity forecasts and other molecular property predictions by transferring data from MD to AI [24].

The fact that the role of DL techniques is a “dark secret” or “black box” must be emphasized [67]. During the training stage, a neural network is only given one particular input with a label. Even the person who created the network may not be aware of what is being examined at the intermediate phases or the reasoning behind the model’s conclusions because the features are not explicitly described. To sum up, a lot of effort has been put towards incorporating AI techniques to speed up the drug discovery and development process, but more effective applications of these techniques will be required before the complete potential of AI in drug discovery and development is achieved [24].

## Conclusions

With the overwhelming increase of clinical data and advancements in ML techniques and especially DL techniques, AI has enhanced the potential in various clinical aspects of CRC. AI algorithms are used for CRC including CRC identification, therapeutic evaluation, survival prediction, etc. However, there is not much literature available on the application of AI in CRC treatment. For better results, data quantity and quality are the important factors to be improved for precise treatment. The rationale behind any DL algorithm’s conclusions is the accurate calculation of the binding affinity of a novel drug candidate, along with the type of treatment to be selected for and individuals whom AI has to advance with preciseness. Despite ground-breaking advancements in AI-infused medication design and research, there is still a long way to go before personalized therapy for cancer patients can be effectively applied. This demonstrates the potential of AI technology along with current limitations.

## Abbreviations

3D: three-dimensional
AI: artificial intelligence
ANN: artificial neural network
CNNs: convolutional neural networks
CRC: colorectal cancer
DD: drug designing
DL: deep learning
Eqn. 1: equation 1
FDA: Food and Drug Administration
FOLFIRI: leucovorin, fluorouracil, and irinotecan
LR: logistic regression
MD: molecular dynamics
ML: machine learning
nCRT: neoadjuvant chemoradiotherapy
R&D: research and development
RF: random forest
RNNs: recurrent neural networks
SVMs: support vector machines

## Declarations

### Copyright

© The Author(s) 2023.
